# Transcriptome analysis reveals molecular targets of erythrocyte invasion phenotype diversity in natural *Plasmodium falciparum* isolates from Cameroon

**DOI:** 10.3389/fpara.2024.1370615

**Published:** 2024-05-24

**Authors:** Ines A. Ngoh, Karim Mane, Jarra Manneh, Fatoumata Bojang, Aminata S. Jawara, Theresia N. Akenji, Damian N. Anong, Umberto D’Alessandro, Alfred Amambua-Ngwa

**Affiliations:** ^1^ Department of Microbiology and Parasitology, University of Bamenda, Bambili, Cameroon; ^2^ Disease Control and Elimination (DCE), Medical Research Council The Gambia Unit at the London School of Hygiene and Tropical Medicine (LSHTM), Fajara, Gambia; ^3^ Wellcome-Medical Research Council (MRC) Cambridge Stem Cell Institute, Cambridge, United Kingdom

**Keywords:** *Plasmodium falciparum*, transcriptome, RBC invasion, phenotype diversity, malaria, vaccine targets, gene expression

## Abstract

Further understanding of the molecular mediators of alternative RBC invasion phenotypes in endemic malaria parasites will support malaria blood-stage vaccine or drug development. This study investigated the prevalence of sialic acid (SA)-dependent and SA-independent RBC invasion pathways in endemic *Plasmodium falciparum* parasites from Cameroon and compared the schizont stage transcriptomes in these two groups to uncover the wider repertoire of transcriptional variation associated with the use of alternative RBC invasion pathway phenotypes. A two-color flow cytometry-based invasion-inhibition assay against RBCs treated with neuraminidase, trypsin, and chymotrypsin and deep RNA sequencing of schizont stages harvested in the first *ex vivo* replication cycle in culture were employed in this investigation. RBC invasion phenotypes were determined for 63 isolates from asymptomatic children with uncomplicated malaria. Approximately 80% of the isolates invaded neuraminidase-treated but not chymotrypsin-treated RBCs, representing SA-independent pathways of RBC invasion. The schizont transcriptome profiles of 16 isolates with invasion phenotypes revealed a total of 5,136 gene transcripts, with 85% of isolates predicted at schizont stages. Two distinct transcriptome profile clusters belonging to SA-dependent and SA-independent parasites were obtained by data reduction with principal component analysis. Differential analysis of gene expression between the two clusters implicated, in addition to the well-characterized adhesins, the upregulation of genes encoding proteins mediating merozoite organelle discharges as well as several conserved, virulent, merozoite-associated, and exported proteins. The latter majority have been shown to have structural and physiological relevance to RBC surface remodeling and immune evasion in malaria and thus have potential as anti-invasion targets.

## 1 Background

Invasion of human red blood cells (RBCs; erythrocytes) by *Plasmodium falciparum* merozoites is a crucial step in malaria disease development. Consequently, molecular players in the RBC invasion constitute important targets for drug and vaccine development (Burns, 2019; Duffy and Gorres, 2020). The process of merozoite invasion of RBCs is a sequence of steps, requiring multiple protein–protein interactions within the merozoite itself and between the merozoites and RBCs. These interactions are underlined by secretions from merozoite organelles, mainly micronemes, rhoptries, and dense granules (Weiss et al., 2015; Cowman et al., 2017). Proteins involved in invasion belong to two classes. These are adhesins or ligands that mediate strong irreversible binding to specific RBC receptors and are secreted earlier in the invasion process and invasins that do not necessarily bind RBC receptors but mediate later steps of invasion and remodel the host RBC membrane (Cowman et al., 2012; Sharma and Chitnis, 2013).

Ligand–receptor interactions are said to determine specific pathways of RBC invasion, with the latter occurring variably and redundantly between parasite populations (Stubbs et al., 2005; Cowman et al., 2017). Two important merozoite adhesin families, *P. falciparum* erythrocyte binding antigens (PfEBA) and reticulocyte binding-like homologues (PfRh), are known to function cooperatively to mediate alternative use of ligand–receptor interactions for RBC invasion (Stubbs et al., 2005; Lopaticki et al., 2011; Tham et al., 2012). The EBA family, comprising EBA-140, EBA-175, EBA-181, EBL-1, and the pseudogene EBA-165, localizes to the micronemes and binds to sialic acid (SA) residues on RBC receptors, including glycophorins (GYP) A, B, and C. On the other hand, the Rh family, comprising Rh1, Rh2a, Rh2b, Rh4, and Rh5, localizes to the rhoptries. Rh1 appears to function earlier than the other *Pf*Rh members and binds to the sialated receptor Y. Rh2a and Rh2b are paralogues, and together with Rh4, they bind non-sialated receptor Z and complement receptor 1 (CR1) peptides. Rh5 is known to be essential, secreted later in invasion, and binds the basigin receptor (Duraisingh et al., 2003; Goel et al., 2003; Triglia et al., 2005, 2009; Tham et al., 2012).

Defining invasion pathways relies on studying invasion into RBCs treated with standard neuraminidase (N) and protease enzymes [trypsin (T) and chymotrypsin (C)] known to cleave parts of the receptor repertoire on the RBC surface on which the ligands bind (Baum et al., 2003). N cleaves SA residues from GYPA, GYPB, and GYPC, as well as receptors E and Y. Trypsin removes GYPA and GYPC, as well as non-sialated CR1. Chymotrypsin removes non-sialated CR1, receptor Z, and band 3. Thus, by defining the use of SA-anchored receptor–ligand interactions, *P. falciparum* invasion pathway phenotypes are described as either SA-dependent or SA-independent.

Nonetheless, current knowledge on the use of SA-dependent or SA-independent pathway phenotypes is limited to variations in allelic composition and transcriptional variation of EBA and Rh adhesins and remains poorly understood, as reviewed by Ararat-Sarria et al. (2019). The molecular mediators of transcriptional variation and the usage of EBA and Rh ligands in alternative ligand–receptor interactions remain to be elucidated. Several studies using quantitative real-time polymerase chain reaction (RTqPCR) have shown that *P. falciparum* can use alternative invasion receptor–ligand pathways by modulating the expression and utilization of EBA and Rh proteins (Gomez-Escobar et al., 2010; Bowyer et al., 2015; Mensah-Brown et al., 2015). While RT-qPCR remains the gold standard for gene expression analysis, parallel cDNA (RNA) sequencing is a more sensitive approach for providing high-resolution measurements of gene expression and for detecting low abundance transcripts in different phenotypes (Sîrbu et al., 2012). Along these lines, this study investigated, with schizont stages harvested during the first *ex vivo* replication cycle *in vitro*, the prevalence of SA-dependent and SA-independent pathways of RBC invasion in *P. falciparum* parasites from two malaria endemic hotspots in Cameroon. Then, the schizont transcriptome profiles of parasites with SA-dependent and SA-independent pathways were compared to uncover the wider repertoire of transcriptional variation (adhesins and invasins) associated with the use of alternative RBC invasion pathway phenotypes. We hypothesized that natural parasites with distinct invasion pathway phenotypes would be driven by the expression of different invasion-related genes.

## 2 Materials and methods

### 2.1 Sample collection and processing

*Plasmodium falciparum* isolates were obtained from asymptomatic children (< 15 years old) attending health facilities in the Ndop and Limbe health districts located in the northwest region (NWR) and southwest region (SWR) of Cameroon, respectively, between 2017 and 2018. The NWR and SWR are known hotspots of malaria transmission in Cameroon and belong to contrasting geo-ecological strata that influence malaria transmission. NWR presents at high altitudes (~1200 m above sea level) with a characteristic savannah highland cold climate, while SWR presents at low altitudes (<10 m above sea level) with a characteristic coastal lowland humid climate. While NWR is considered hypoendemic, SWR is considered holoendemic for malaria transmission (Massoda Tonye et al., 2018).

The study protocols were approved by the Institutional Review Board of the Faculty of Health Science, University of Buea (2017/008/UB/SG/IRB/FHS). At least 2 mL of blood was collected from the study participants with the informed consent of a legal guardian or the assent of older children. Children who reported being on antimalarials were excluded from the study. After microscopic diagnosis in the laboratory, infected RBCs of blood samples with high parasite density (>1% parasitemia, since samples had to be cryopreserved) were separated from leucocytes via centrifugation and washed twice with RPMI1640 medium. The resultant packed RBCs were mixed with three parts of a 57% glycerolyte solution in 500-µL vials and transported in liquid nitrogen to the MRC Unit The Gambia, for further experiments.

### 2.2 Parasite culture and schizont enrichment

Cryopreserved clinical isolates and laboratory-adapted *Plasmodium falciparum* 3D7 and Dd2 strains (serving as controls) were thawed and subjected to *in vitro* culture. All parasites were cultured at 37°C in complete RPMI-1640 medium (supplemented with 0.5% AlbuMAX II (Gibco, USA) for laboratory strains and with 20% heat-inactivated human AB serum for clinical isolates). Cultures were maintained at 2% hematocrit (HCT) in 10-mL culture flasks using human group O^+^ RBCs from a single donor and incubated in a gas mixture of 1% O_2_, 5% CO_2_, and 94% N_2_.

Schizont stage enrichment proceeded with maintaining parasites in cultures for up to 48 h with close monitoring every 6 h for parasite stage development using light microscopy of Giemsa-stained thin smears. When most infected erythrocytes were segmented with eight or more visible merozoites, parasite cultures were harvested. Approximately 500 µL of the harvested parasite culture was aliquoted for use in setting up invasion-inhibition assays. The remaining culture volume was centrifuged, and the recovered pellet was layered on 3 mL of 70% Percoll (Sigma-Aldrich)/phosphate buffered saline (PBS) density (v/v) solution and centrifuged at 800g for 10 min. After which, the supernatant containing the schizont-concentrate was washed twice in complete RPMI, mixed with a 4× volume of TRIzol^®^ reagent (Ambion, USA), and stored at −80°C until RNA extraction. While schizont enrichment was done in the first *ex vivo* replication cycle for field isolates, laboratory strains, on the other hand, were subjected to one growth cycle and then tightly synchronized to ~90% ring stages by treating twice with 5% w/v D-sorbitol for 5 min prior to schizont enrichment in the second cycle.

### 2.3 Erythrocyte invasion-inhibition assays for determining invasion phenotypes

**Figure 1:**
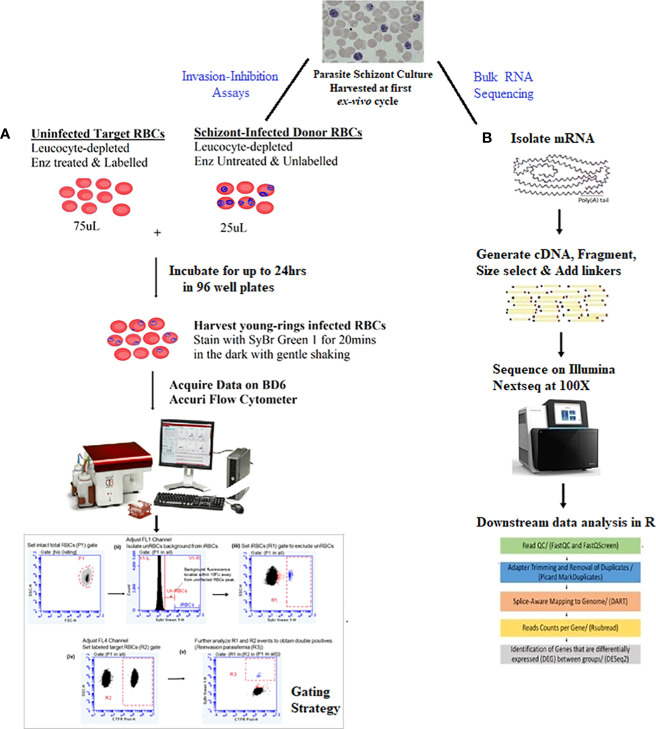
Summary of study protocol.The study proceeded with schizont stages isolated in the first *ex-vivo* replicative cycle of in-vitro culture of the isolates. **(A)** Two-color flow cytometry-based invasion-inhibition assay protocol together with the gating strategy for quantifying reinvasion parasitemia and determining erythrocyte invasion pathway phenotypes of isolates. Unlabeled schizont-infected RBCs (donor RBCs) were incubated with CTFR labelled enzyme-treated O^+^ uninfected RBCs (target RBCs) from a single donor for up to 24hrs. After which cells were harvested and stained with SyBr Green 1 for 20mins. Assays were run in a 1:3 ratio of donor: target RBCs in 100µL reaction volume in 96 well plates. **(B)** RNA sequencing protocols for obtaining and comparing transcriptome profiles of isolates. Schizont stage mRNA was isolated, fragmented and copied into stable double stranded DNA (ds-cDNA). The ds-cDNA was size selected, adaptor-ligated and sequenced using high-throughput, short-read sequencing on an Illumina Nextseq instrument. Resultant reads were then subjected to downstream bioinformatics processing with R statistical packages.

Erythrocyte invasion-inhibition assays proceeded with a two-color flow cytometry (FCM) protocol using schizont-infected RBCs (donor RBCs) and fluorescently labeled enzyme-treated O^+^ uninfected RBCs (target RBCs) from a single donor, as reported elsewhere (Ngoh et al., 2020). A schematic of the FCM invasion-inhibition assay protocol is shown in (**Figure 1A**).

Briefly, freshly washed target O+ RBCs were fluorescently labeled with 10-µM cell trace far red (CTFR) at 37°C for 2 h on a rotating wheel. After which, they were washed and treated with the following enzyme concentrations in corresponding labeled tubes: 33.35 mU/mL of N (2 U/mL, Cat N6514, Sigma), 1 mg/mL of T (high T) (Sigma, Cat T9935), 1 mg/mL of C (high C) (Sigma, Cat C4129), 33.35 mU/mL of N + 0.67 mg/mL of T (N + low T), 1 mg/mL of C + 0.67 mg/mL of T (high C + low T), and 1 mg/mL of T + 0.67 mg/mL of C (high T + low C). A positive control tube with all enzymes’ treatments (N + C + T) and a negative control tube with no enzyme treatment (RPMI) were included. Given that each enzyme cleaves a specific set of receptors, enzyme combinations were added to study which ligand–receptor pathways are collectively used by parasites, and by so doing, characterize the dominant receptor pathways of the isolates.

Assays were set up in 96-well plates at 2% HCT in 100-μL assay volumes. For each sample, 25-μL donor RBCs were incubated with 75-μL target RBCs (i.e., a 1:3 plating ratio of donor to target RBCs). Donor RBCs were diluted to an initial parasitemia of 0.1% prior to assay set-up. Each assay was run in triplicate wells. Cells were incubated for up to 24 h until reinvasion occurred with the emergence of post-8-h young rings. After which, cultures were harvested, washed, and stained with 0.0002 U/mL of SyBr Green I for 20 min in a dark room. Flow cytometry data acquisition, visualization, and analysis were performed on a BD Accuri™ C6 flow cytometer and BD6 Csampler software (BD Biosciences, Oxford, UK). Details of the FCM gating strategy to obtain parasitemia after reinvasion have been added as a supplement (**Supplementary Methods**). The percentage inhibition of parasite invasion by enzyme treatment was calculated using a previously described formula (Bowyer et al., 2015). Parasites with invasion inhibition greater than 50% were considered sensitive (s) to the enzyme treatment, while those with invasion inhibition lower than 50% were considered resistant (r) to the enzyme treatment.

### 2.4 RNA extraction, library preparation, and sequencing

Frozen schizont concentrates mixed with TRIzol (of clinical isolates + 3D7 and Dd2 controls) were thawed at room temperature, and the total RNA of each schizont concentrate was extracted with RNeasy mini columns (QIAGEN) according to the manufacturer’s protocol. A schematic of the RNA sequencing protocol is shown in (**Figure 1B**). DNA was removed by DNase I digestion on RNeasy mini columns (QIAGEN, Germany). The total RNA was eluted in 50-μL RNase-free H_2_O and stored at −80°C until library preparation. Prior to storage, the quantity of the extracted total RNA was checked using the Qubit High Sensitivity RNA Assay (Qubit 3.0 Fluorimeter, Life Technologies, USA). For samples containing at least 200 ng of total extracted RNA (that is > 4 ng/µL of eluted volume), RNA quality was checked on the tape station (Agilent 2100 Bioanalyzer, Germany) using RNA 6000 Nano Kit [reagents and chips (**Supplementary Figure 1**)]. Sequencing library preparation proceeded with an initial input of 150 ng of total RNA per sample with an RNA integrity number (RIN) or score of at least 7. Messenger RNA libraries were prepared using the NEBNext^®^ Poly(A) mRNA Magnetic Isolation Module (E7490) and NEBNext^®^ Ultra™ II RNA Library Prep Kit for Illumina^®^ (E7770, E7775) as per the manufacturer’s protocols, New England Biolabs in Germany. Library quality and molarity were determined, respectively, on the Agilent Bioanalyzer using DNA 1000 reagents and chips (Agilent 2100 Bioanalyzer, Germany) and by a real-time PCR system using a KAPA Universal library quantification kit (Roche Diagnostics Limited, Switzerland). Library concentrations were adjusted for library size, then pooled at 12–15 pM concentrations and sequenced for 100X coverage per isolate with 2 × 75 cycles on the Illumina Nextseq550 (Illumina, San Diego, CA, USA) platform at the MRC Unit The Gambia genomics platform. Note that due to the limitations of our multiplex assay, Dd2 was not sequenced at the same time as 3D7 and the clinical isolates that were sequenced in one run. Rather, it was sequenced under the same conditions and with the same technology as part of another dual transcriptome experiment in the same laboratory (unpublished data).

### 2.5 Bioinformatics and RNAseq data analysis

Initial processing and quality assessment of raw paired-end fastQ files involved base quality checks and de-multiplexing of indexed reads using FastQC v11.9 from Babraham Bioinformatics (http://www.bioinformatics.babraham.ac.uk/projects/fastqc/). This was followed by trimming of adapter sequences and filtering of low-quality reads (<36 base pairs) using TrimGalore v0.4.1 tools with the parameter-illumina from Babraham Bioinformatics (http://www.bioinformatics.babraham.ac.uk/projects/trim_galore/). The filtered reads were assembled by alignment to the *P. falciparum* 3D7 v3 reference genome using the division-based alignment for RNA-Seq transcripts (DART) algorithm in R (Lin and Hsu, 2018). Reads originating from PCR duplication in the 3D7-aligned reads were removed using the Picard version 2.9.1 “MarkDuplicates” function (https://broadinstitute.github.io/picard/). The genome-wide coverage of each sample was determined using Qualimap2 (Okonechnikov et al., 2016). Mapped reads were then converted to BAM files using SAM tools (Li et al., 2009). The proportion of genomic DNA (gDNA) contamination in our RNAseq library was visually assessed on multi-exon genes in Artemis (Carver et al., 2012) to ensure that reads did not extend into introns. Raw read counts of each gene (transcript abundance) were estimated with the Rsubread package in R (Liao et al., 2019) using a *P. falciparum* 3D7 reference genome annotation file that had masked out extremely polymorphic gene regions, duplicated genes, and the *var*, *rifin*, and *stevor* large sub-telomeric gene families (Tarr et al., 2018). The proportion of parasite developmental stages in the transcriptome data was investigated using two approaches (**Supplementary Methods**). Raw read counts were normalized for variations in sequencing depth and gene length, and the quality of the normalization was checked on a dispersion plot.

To investigate sample clustering and identify any outliers in the normalized gene expression data prior to differentially expressed genes (DEG) analysis, hierarchical clustering and principal component analysis (PCA) were performed in DESeq2. A heat map of the hierarchical clustering of samples was produced using the Pheat Package of DESeq2. The heat map was constructed from a similarity matrix of sample-to-sample Cook’s distances computed from the regularized log (rlog) transformed count data. An associated metadata of the invasion pathway profile (SA-dependent versus SA-independent) and a sample origin (NWR versus SWR) as covariables were included. A PCA plot was also constructed from the rlog-transformed count data. To increase the probability of identifying differentially transcribed genes between groups, a false discovery rate (FDR) = 0.05, a threshold of genes with a log2 fold change ≥2.0 (more than a four-fold difference), and a Benjamini–Hochberg adjusted *p*-value < 0.01 were applied. DEG data were visualized with enhanced volcano plots in R (rama-rhoptry associated membrane antigen). Enriched terms for specific biological processes, cellular functions, cellular components, and biological pathways in the differentially highly expressed genes were investigated using the gene ontology (GO) (Ashburner et al., 2000) and Kyoto Encyclopedia of Genes and Genomes (KEGG) pathway (Kanehisa and Goto, 2000) plugins in the PlasmoDB webserver release 35 (Aurrecoechea et al., 2009). A Benjamini–Hochberg adjusted *p*-value < 0.05 was applied to the test for statistical significance.

### 2.6 Reverse transcription-quantitative PCR validation

RT-qPCR was performed to confirm the presence and transcript abundance of a select set of differentially expressed genes (*eba140, eba175*, *Rh2b*, *Rh4, surf4.2*, *surf8.2*, *msp6*, *clag3.1*, and *mspdbl2*) obtained with RNA-seq. *Rh5* was not significantly differentially expressed but was added to the tested panel of genes. Genes were selected based on their merozoite surface attachment or secretory potential and the availability of primers (**Supplementary Table 1**) at the time of study. Briefly, 150–500 ng of total extracted RNA from schizont stage preparations used in RNAseq was reverse transcribed using the QuantiTect^®^ Reverse Transcription Kit (QIAGEN) with an optimized mix of 250-ng oligo-dTs and random primers per 20-μL reaction volume. The cDNA was quantified with a Qubit High Sensitivity dsDNA Assay (Qubit 3.0 Fluorimeter, Life Technologies). Quantitative real-time PCR was carried out in 10-μL reaction volumes in 96-well plates using a 2X QuantiTect^®^ SYBR Green RT-PCR Master Mix (QIAGEN) with a Bio-Rad PCR instrument (Applied Biosystems, California, USA). The cycling conditions included initial activation and denaturation at 95°C for 30 s, annealing at 50°C for 40 s, and extension at 65°C for 50 s. The RT-qPCR transcript levels of each individual gene were normalized against transcript levels of the housekeeping gene, *gapdh* (glyceraldehyde 3-phosphate dehydrogenase), and a schizont stage control gene, *ama1* (apical merozoite antigen 1). The average of the two normalizations was retained for comparisons.

### 2.7 Statistical analysis

Plots and statistical analysis of invasion phenotype and RT-qPCR data were done on GraphPad version 10.01. Invasion phenotype data were not normally distributed and were thus analyzed using non-parametric tests. The Kruskal–Wallis test was used to test for significant pairwise differences in percentage invasion inhibition between enzyme treatments, with Bonferroni correction. Statistical significance was generally considered at a *p*-value < 0.05.

## 3 Results

### 3.1 Clinical and demographic characteristics of the study participants

The clinical and demographic parameters of the study participants are shown in **Table 1**. A total of 127 samples (including 68 from SWR and 59 from NWR) were thawed and subjected to *in vitro* culture at the MRC Unit The Gambia at the LSHTM malaria cell culture laboratory. The first *ex vivo* cycle invasion pathways for all enzyme treatments were successfully obtained for 63 (49.6%) of the 127 cultured isolates, including 54 (85.7%) from SWR and 9 (14.2%) from NWR. The remaining 64 samples that could not be phenotyped had either been lysed and had no red cell pellet upon thawing (12 isolates) or had poor parasite growth in culture (52 isolates). No statistically significant difference (*p*= 0.086, using the Kruskal–Wallis test) was observed in the sex distribution, ages, or parasite density of the study participants from the two geo-ecological regions. A slightly higher proportion (52% vs. 48%) of male children compared to female children was recruited, and the ages of the study participants ranged from 1.8 to 12 years and 2.6 to 15 years in the NWR and SWR, respectively. No significant differences were observed in the sex and ages of the participants from both regions (p = 0.496 and p = 0.863, respectively, using the Kruskal–Wallis test). A higher median parasite density was observed in the coastal lowland areas of the SWR (3.7 ± 0.016) than in the highland areas of the NWR (2.6 ± 0.028), mirroring the relative transmission intensities in the areas. However, this difference did not attain statistical significance at α = 0.05 (p = 0.076). The parasite densities at the two health districts were expected not to differ significantly because sample collection at the hospitals was biased toward high parasitemia samples for cryopreservation. RNA sequencing was performed on pooled cDNA (messenger RNA) from 16 schizont preparations (including 10 from SWR and 6 from NWR) with invasion phenotypes, alongside one 3D7 control sample. The remainder of the samples (47) that could not be sequenced had poor quality RNA or insufficient RNA input (<150 ng) for library preparation. Again, no statistically significant difference (*p*= 0.086, using the Kruskal–Wallis test) was observed in the sex distribution and ages of the study participants from the two geographical regions.

**Table 1:** Clinical and demographic characteristics of the study participants.

Sample Characteristic	Cameroon		Combined	P value ^a^
	SWR	NWR		
Thawed	68	59	127	…
Invasion phenotyped	54 (85.7%)	9 (14.2%)	63	
Sex (male, female)	29, 25	4, 5	33, 30	0.496
Age (years) (range, median ± SD)	2.5 to 15, 4.6 ± 0.081	1.5 to 12, 5.9 ± 0.072	1.5 to 15, 5.3 ± 0.029	0.863
Parasitemia (%) upon thawing (median ± SD)	3.7 ± 0.016	2.6 ± 0.028	3.2 ± 0.104	0.076
RNA sequenced	10 (62.5%)	06 (37.5%)	16	
Sex (male, female)	5, 5	4, 2	9, 7	0.283
Age (years) (median ± SD)	7.9 ± 0.08	6.8 ± 0.08	6.7 ± 0.2	0.057

P values were obtained using the Kruskal–Wallis test. Plasmodium falciparum isolates were obtained from asymptomatic children (< 15 years old) attending health facilities in the Ndop and Limbe health districts located in the northwest region (NWR) and southwest region (SWR) of Cameroon, respectively.

### 3.2 Erythrocyte invasion phenotypes of Cameroonian *P. falciparum* clinical isolates

**Figure 2:**
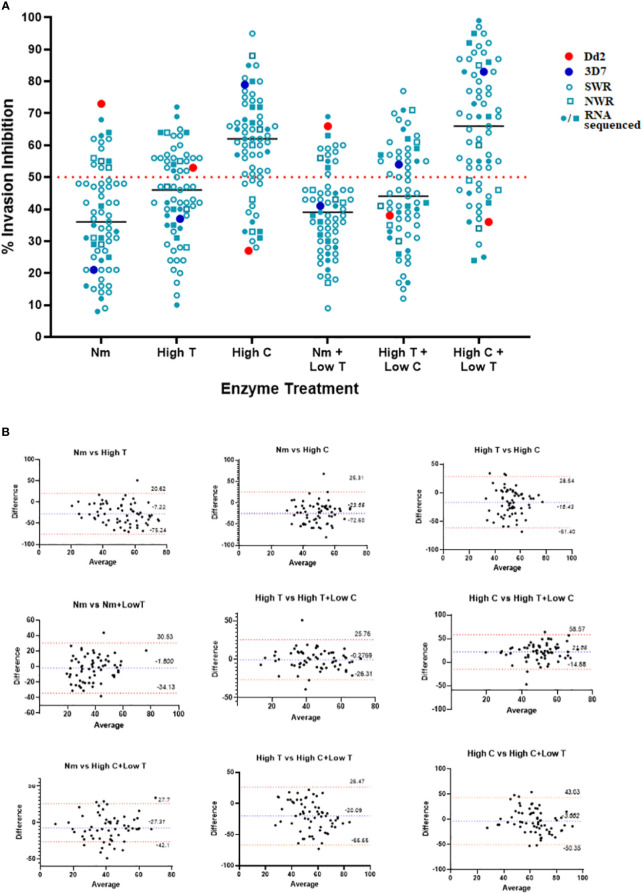
Erythrocyte invasion phenotypes of *Plasmodium falciparum* isolates from Cameroon. **(A)** Scatter plot of percent (%) invasion inhibition by different enzyme treatments and enzyme treatment combinations. Enzyme treatments were combined to appreciate their combined effect on RBC-receptor deficiency and invasion inhibition. Target RBCs were treated with 33.35 mU/mL of neuraminidase (N), 1 mg/mL of trypsin (high T), 1 mg/mL of chymotrypsin (high C), 33.35 mU/mL of neuraminidase + 0.67 mg/mL of T (N + low T), 1 mg/mL of C + 0.67 mg/mL of T (high C + low T), and 1 mg/mL of T + 0.67 mg/mL of C (high T + low C). Given that each enzyme cleaves a specific set of receptors, the use of combined enzyme treatments was also employed to study which ligand–receptor pathways are collectively used by parasites, and by so doing, characterize the dominant receptor pathways of the isolates. The dotted line at 50% represents the cut-off for establishing invasion-inhibition efficiency. Parasites with invasion inhibition >50% were considered sensitive (s) to the enzyme treatment, while those with invasion inhibition <50% were considered resistant (r) to the enzyme treatment (Baum et al., 2003). Assays were set up in 96-well plates at 2% HCT in 100-mL assay volumes with an unlabeled donor-labeled target RBC ratio of 1:3 (i.e., 25 uL:75 uL) per assay well. **(B)** Bland–Altman plots comparing (%) invasion inhibition between single enzyme treatments and between single and combined enzyme treatments. The bias **(b)** and 95% confidence interval for both the lower and upper limits of agreement are shown for each pair of comparisons. Data points are the means of triplicate assay wells.

Our invasion-inhibition assays recapitulated known invasion pathway phenotypes for the laboratory controls: SA-independent for 3D7 (21% and 79% invasion inhibition for N and C, respectively) and SA-dependent for Dd2 (73% and 27% invasion inhibition for N and C, respectively) (**Figure 2A**). Overall, the invasion phenotypes of Cameroonian isolates were dominated by sialic acid-independent pathways of RBC invasion. This was evident from N treatment having a relatively lower inhibitory effect on parasite invasion (range: 6%–68%, median: 36%) than high T (range: 10%–72%, median: 46%) or high C treatment (range: 28%–95%, median: 62%) (**Figure 2A**). Based on the enzyme sensitivity (s)/resistance (r) data (**Supplementary Figure 2**), ~80% of isolates (50/63) had a neuraminidase-resistant (Nr) and chymotrypsin-sensitive (Cs) profile. This included NrTsCs (29/63) and NrTrCs (21/63), which are characteristic of SA-independent receptor–ligand interactions. The remaining (~20%) isolates had neuraminidase-sensitive (Ns), NsTsCr (7/63), NsTrCs (3/63), and NsTrCr (3/63), which are characteristic of SA-independent receptor–ligand interactions. Similar trends were observed between NWR and SWR isolates, although the biased sample sizes (9 NWR against 54 SWR) did not allow for statistically significant comparisons.

Comparisons between combined and single enzyme treatments further reiterated the dominance of SA-independent receptor-ligand interactions in the Cameroonian isolates. The inhibitory effect of high C + low T (range: 24%–98%, median: 68%), which should cleave more CR1 and Z as well as some GYPA and GYPC on the RBC surface, was greater than the individual effect of high C alone or low T alone, mirroring the effect on 3D7. Similarly, high T + low C (range: 13%–71%, median: 48%), which should cleave GYPA and GYPC as well as more CR1 and Z, was less than the effect of high C but greater than the individual effect of high T alone. N + low T (range: 9%–61%, median: 41%), on the other hand, was sandwiched between the individual effects of N and high T (**Figure 2A**). These results were corroborated by Bland–Altman comparisons of single and combined enzyme treatments. Good levels of agreement were observed between invasion inhibition obtained with single enzymes and between single and combined enzymes that cleave similar receptors on the RBC surface. The observed mean differences (bias) are as follows: N vs. high T = −7.22, N vs. high C = −23.05, high T vs. high C = −10.43, N vs. N + low T = −1.8, N vs. high C + low T = −27.05, high T vs. high T + low C = −0.27, high C vs. high C + low T = −3.662, and high T vs. high C + low T = −20.09 (**Figure 2B**).

### 3.3 Schizont stage gene expression profiles and differentially expressed genes in parasites using alternative pathways of RBC invasion

**Figure 3:**
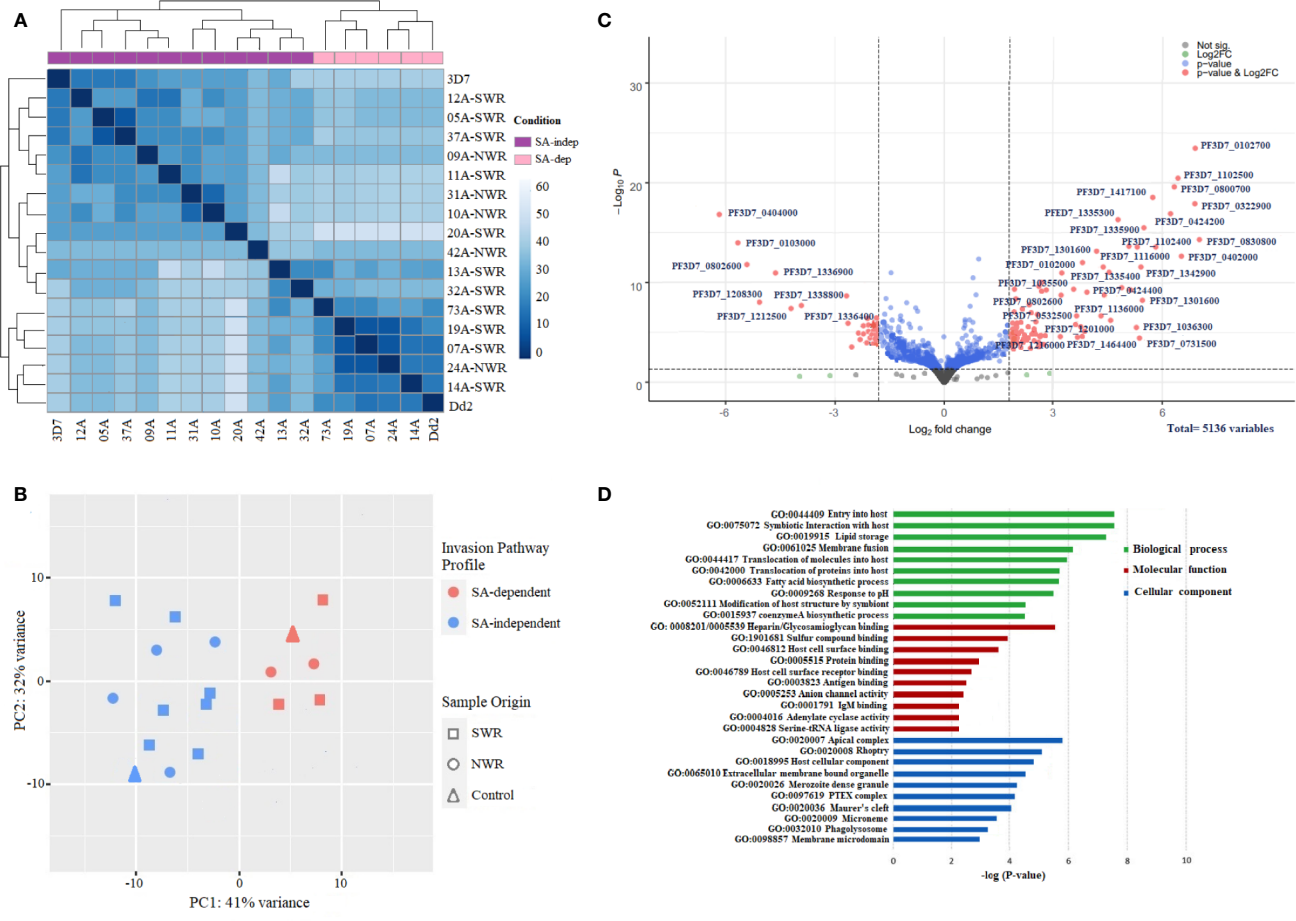
Gene expression profiles and differential expression of genes between sialic acid (SA)-dependent and SA-independent *Plasmodium falciparum* isolates from Cameroon. **(A)** Heat map of the hierarchical clustering of samples into two distinct groups of SA-dependent and SA-independent parasites. The heat map was constructed from a similarity matrix of sample-to-sample Cook’s distances, computed from the regularized log (rlog) transformed count data with an associated metadata of invasion pathway profile (SA-dependent versus SA-independent) and a sample origin (NWR versus SWR) as covariables. **(B)** Scatter plot of data reduction with PCA clustering samples on PC1. The PCA plot was constructed from the regularized log (rlog) transformed count data. **(C)** Volcano plot of significantly differentially expressed genes (DEGs) between sialic acid-dependent and sialic acid-independent isolates. Each dot represents one gene and is displayed according to the log_2_ fold change in expression (x-axis) and the statistical significance of the association (y-axis, in –log10 of the *p*-value). A false discovery rate, FDR = 0.05, and an adjusted *p*-value < 0.01 were applied. Red dots indicate differentially expressed genes with an absolute log_2_ fold change >2, with high expression (right) and low expression genes (left). Green dots are non-significant DEGs. Gene IDs of the top significant upregulated and downregulated DE genes are shown. **(D)** Bar plot showing the top 10 significantly (p < 0.05) enriched gene ontology (GO) terms for the significant upregulated differentially expressed genes between SA-dependent and SA-independent isolates from Cameroon. Green, red, and blue bars represent enriched features for biological processes, molecular function, and cellular components, respectively, with the corresponding negative log10 of adjusted *p*-values. The analysis was performed using the Gene Ontology (GO) and Kyoto Encyclopedia of Genes and Genomes (KEGG) pathway plugins in the PlasmoDB webserver release 35.

The RNA sequencing run generated an average of ~411 million paired-end reads, with wide variation in sequencing depth (mean: 78.89X, range: 71.31X–89.13X) across all the genes in each isolate (**Supplementary Table 2**). The total number of raw reads per sample ranged from 18 to 33 million. The percentage of reads that mapped onto the *P. falciparum* 3D7 reference genome was averagely 89.63% across all samples, from which a total of 5,136 *Plasmodium falciparum* gene transcripts were counted (**Supplementary Table 3**). Gene counts were adequately normalized for variations in sequencing depth and gene length with a near-zero coefficient of variation line obtained with a dispersion plot (**Supplementary Figure 2**). Deconvolution of parasite developmental stage proportions in the transcriptome data identified all samples to have >82% of parasites at late-stage schizonts [> 40 h post-invasion (hpi)] (**Supplementary Figure 3** and **Supplementary Table 4**), implying that the schizont enrichment of parasite cultures had been largely successful, with all samples qualifying for subsequent differential expression analysis. Unsupervised clustering revealed that the invasion pathway profile, to some extent (and not sample-geo-ecological origin), appeared to be the main source of variation in the gene expression data of the samples, producing distinct SA-dependent and SA-independent clades on the heat map of hierarchical clustering (**Figure 3A**) and PCA plot (**Figure 3B**). Most isolates broadly clustered together almost evenly along principal component 1 (PC1), which accounted for 42% of the variance.

Focusing on genes in the schizont transcriptome profiles of SA-dependent and SA-independent parasites with log2 fold change >2 (more than four-fold difference) and adjusted *P* value < 0.01, we identified 127 genes to be significantly differentially expressed between the two groups (**Figure 3C**; **Supplementary Table 5**), with 84 upregulated genes and 43 downregulated genes. Among the significantly upregulated DEGs were genes encoding merozoite adhesins: *eba140*, *eba175, eba 181*, and *rh1* with higher expression in SA-dependent parasites, against *rh2a, rh2b*, and *rh4* with higher expression in SA-independent parasites. *Rh5* was not significantly differentially expressed between the two groups. A good number of invasin genes encoding several merozoite-associated, exported, and virulent proteins were significantly upregulated in SA-independent parasites relative to SA-dependent parasites. These included rhoptry-associated protein (*rap1*), merozoite-specific thrombospondin-related anonymous protein (*mtrap*); rhoptry neck (*ron4*); *Plasmodium* helical interspersed sub-telomeric (*phista, phistb*, and *phistc*); exported protein family (*epf1*–*epf4*), ring exported protein (*rex1*), ring erythrocyte surface antigen (*resa*); rhoptry associated membrane antigen (RAMA) *Plasmodium* translocon of exported proteins (*ptex150, exp2*); cytoadherence linked antigens (*clag2, clag 3.1*, and *clag3.2*); surface-associated interspersed proteins (*surf1.2, surf4.1, surf4.2, surf8.2* and *surf8.3);* and merozoite surface proteins (*msp6* and *dblmsp2*). Other genes encoding proteins shown to be involved in signaling microneme secretions of EBA ligands like calcium-dependent protein kinase (*cdpk2* and *cdpk5*), phosphoinositide-binding protein (ph2), and double C2-like domain-containing protein (*doc*) were significantly upregulated in SA-dependent parasites relative to SA-independent parasites. Additionally, a good number of genes encoding transcription factors (*apiap2* and *ap2-g2)*, as well as several conserved proteins of unknown function, had significantly higher expression in SA-independent parasites. GO enrichment analysis on all significantly upregulated DE genes showed numerous enriched terms for *biological processes*, *molecular function*, and *cellular components* at *p* < 0.05 (**Figure 3D**; **Supplementary Table 6**). Among the top 10 significantly enriched terms for biological processes were host cell entry, symbiotic interaction, protein translocation, lipid storage, and membrane fusion; for molecular function, heparin binding, glycosaminoglycan binding, host cell surface binding, antigen and antibody binding; and for cellular components, apical complex, rhoptry, dense granule, PTEX complex, Maurer’s cleft, and microneme.

### 3.4 RT-qPCR validation of upregulated differentially expressed genes

**Figure 4:**
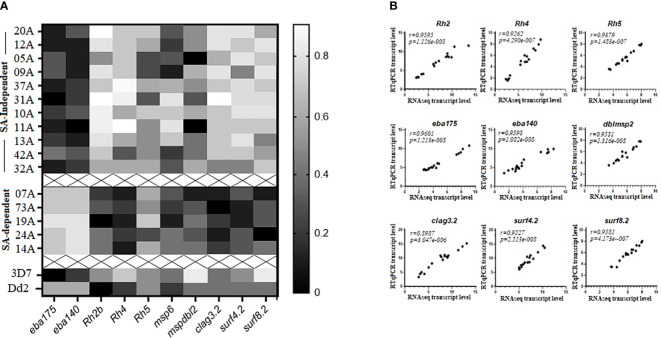
RTq-PCR validation of selected differentially expressed genes (DEGs) between sialic acid (SA)-dependent and SA-independent isolates from Cameroon. **(A)** Heat map of transcript abundance estimates of selected upregulated DEGs (*eba175, eba140, Rh2b, Rh4, Rh5, clag3.2, msp6, mspdbl2, surf4.2*, and *surf8.2)* in schizont stage preparations for 11 clinical isolates using sialic acid-independent pathways of RBC invasion (top), 5 clinical isolates using sialic acid-dependent pathways of RBC invasion cluster 2 (middle), as well as 3D7 and Dd2 laboratory-adapted isolates (bottom). **(B)** Significantly high Spearman’s rank correlation coefficients were observed between RNAseq and RTqPCR-determined log2 transformed transcript abundance estimates for a select set of upregulated DEGs. The RT-qPCR transcript levels of each gene were normalized against transcript levels of the housekeeping gene, *gapdh* (glyceraldehyde 3-phosphate dehydrogenase), and a schizont stage control gene, *ama1* (apical merozoite antigen 1). The average of the two normalizations was retained for comparisons.

RT-qPCR confirmed RNAseq transcript abundance estimates of *eba175, eba140, Rh2b, Rh4, clag3.2, msp6, mspdbl2, surf4.2*, and *surf8.2* that were in the top quartile of upregulated DEGs (**Figure 4A**). *Rh5* was not differentially expressed but was added to the analysis. Expression of *Rh5* and *msp6* was not significantly different between the two clusters of isolates and could thus be considered as part of the “schizont stage gene expression signatures.” In addition, strong positive and highly significant Spearman’s rank correlation coefficients (*r)* were observed between the log2 transformed transcript abundance estimates obtained with RT-qPCR and RNAseq and ranged from *r*=0.898 for the *clag3.2* gene to *r*=0.96 for the *eba175* gene (**Figure 4B**). This was supported by Bland-Altman comparisons of the log2 transformed transcript levels obtained by RT-qPCR and RNAseq measurements of gene expression for each gene set, which revealed good agreement levels between the two measures. The mean differences (bias) observed were as follows: eba 175 = −2.028, eba 140 = −7.206, Rh2b = −7.667, Rh4 = −4.5, Rh5 = −2.5, surf 4.2 = −1.667, surf 8.2 = 2.808, clag 3.2 = −3.694, and dblmsp2 = −8.3 (**Supplementary Figure 5**).

## 4 Discussions

This study characterized the prevalence of SA-dependent and SA-independent pathways of RBC invasion in *P. falciparum* parasites from two malaria endemic hotspots in Cameroon and then compared schizont stage transcriptome variations associated with the use of SA-dependent and SA-independent RBC invasion pathways to uncover the wider repertoire of molecular mediators (adhesin and invasin proteins) of alternative ligand–receptor pathways of RBC invasion.

Overall, Cameroonian isolates showed a dominance of SA-independent RBC invasion pathways. This was consistent with previous reports of other endemic African parasites (Deans et al., 2007; Bowyer et al., 2015; Mensah-Brown et al., 2015). Approximately 80% of the isolates displayed neuraminidase-resistant (Nr) and chymotrypsin-sensitive (Cs) profiles that are typically used by 3D7, HB3, and FVO5 *P. falciparum* strains and have a characteristic SA-independent RBC invasion phenotype mediated by Rh4-CR1, Rh2b-Z, and MSP1-band 3 ligand–receptor interactions (Goel et al., 2003; Triglia et al., 2009; Tham et al., 2012). The remaining (~20%) isolates displayed neuraminidase-sensitive (Ns) and chymotrypsin-resistant (Cr) profiles typically used by *P. falciparum* Dd2 and W2mef strains and have a characteristic SA-dependent invasion pathway phenotype that is mediated by EBA175-GYPA, EBA140-GYPC, and Rh1-Y ligand-receptor interactions (Gilberger et al., 2003; Triglia et al., 2005).

Given that each enzyme cleaves a specific set of receptors, the use of combined enzyme treatments was also implored to study which ligand–receptor pathways are collectively used by parasites, and by so doing, characterize the dominant receptor pathways of the isolates. For example, N cleaves the sialated GYPA, GYPB, and GYPC. T cleaves GYPA, GYPC, and non-sialated CR1. C cleaves non-sialated CR1, receptor Z, and band 3. A combination of N + low T, for example, should cleave GYPB, more GYPA and GYPC + some CR1 and Z on the RBC surface. Thus, we will expect the inhibitory effect of N + low Ton parasites that are dependent on SA residues for invasion like Dd2 to be greater when compared to N or T alone. On the other hand, a combination of high C + low T will cleave more CR1 and Z + some GYPA and GYPC. Thus, we will expect the inhibitory effect of high C + low T on parasites that are dependent on non-sialated receptors like 3D7 to be greater than that of high C or high T alone. Our findings revealed that the Cameroonian isolates mirrored 3D7 and not Dd2, reinforcing their dominant use of SA-independent pathways. In addition, data from Bland–Altman comparisons revealed strong level agreements between N and N + low T as well as between high C and high C + low T measurement of invasion phenotypes, suggesting that these enzyme combinations can be incorporated in future studies on defining RBC invasion pathway phenotypes of isolates.

Interestingly, and concurrent with the functional data, transcriptome analysis produced distinct SA-dependent and SA-independent parasite clusters with significantly higher expression of *Pfeba 140, Pfeba 181*, and *PfRh1* genes in SA-dependent parasites relative to SA-independent parasites and significantly higher expression of *PfRh2a, PfRh2b*, and *PfRh4* in SA-independent isolates relative to SA-dependent isolates. This was not surprising since invasion pathway phenotypes have strongly been associated with differential expression of merozoite adhesins belonging to *P. falciparum eba* and *Rh* gene families (Lopaticki et al., 2011; Bowyer et al., 2015; Mensah-Brown et al., 2015; Campino et al., 2018) that are secreted by micronemes and rhoptries, respectively, and function during the initial binding stages of invasion (Cowman et al., 2017). In addition, *PfRh2a* and *PfRh2b* are closely related proteins, and the upregulation of *PfRh2b* has been associated with increased sensitivity to chymotrypsin treatment (Campino et al., 2018). In contrast, adhesins like *ama1* and *PfRh5* that function during the latter binding stages of invasion (Cowman et al., 2017) were not significantly differentially expressed. Thus confirming the essential role of these proteins and suggesting that adhesins that function during the initial binding stages of invasion are selected in schizont stages prior to merozoite entry into RBCs.

In addition to adhesins, several genes encoding merozoite invasins [non-receptor binding proteins mediating the invasion process (Cowman et al., 2017)] had significantly higher expression in SA-independent parasites relative to SA-dependent parasites. In order for parasites to survive and multiply in circulation, they remodel the host RBC membrane, making it more permeable for nutrient absorption and toxic waste excretion in the bloodstream (Counihan et al., 2013). It also remodels the RBC surface for increased cytoadherence, cell-to-cell communication, and stress resistance (Sisquella et al., 2017) in a bid to evade host immunity. RBC remodeling integrates several exported, secreted, merozoite-associated, and virulence-associated proteins reported here. PTEX is the translocon for protein export. It comprises three core proteins, EXP2, HSP101, and PTEX150, which were upregulated in this study. EXP2 has been shown to be the dominant membrane-associated component in schizonts (Bullen et al., 2012). PTEX150, EXP2, and RESA are secreted from merozoite dense granules (Bullen et al., 2012), and together with PHIST proteins, they function to facilitate the translocation of several merozoite proteins containing a *Plasmodium* export element (PEXEL) and destined for export onto the RBC surface (Chisholm et al., 2016; Morita et al., 2018). The significance of their upregulation of PTEX in SA-independent isolates is not clear, but we speculate that the involvement of unknown protein signaling between PTEX trafficking of virulent proteins and rhoptry releases in SA-independent parasites exists and is necessary for efficient alternative pathways of RBC entry and immune evasion.

Additionally, genes encoding several export and virulent protein families, including SURFIN, EPF, and CLAG proteins, were implicated. SURFINs have been localized to the merozoite surface with putative roles in erythrocyte invasion and/or immune evasion (Winter et al., 2005). Recent evidence from immunoprecipitation (IP) followed by mass spectrometry (MS) studies revealed that SURFIN 4.2 interacts with rhoptry neck protein 4 (RON4) and glutamate-rich protein (GLURP) to form a complex termed SURGE (SURFIN4.2-RON4-GLURP), which is thought to be necessary for RBC invasion (Quintana et al., 2018). Remarkably, these proteins were all upregulated in the SA-independent group. EPF proteins are Maurer’s cleft-associated proteins, and their increased expression results in efficient merozoite releases (Mbengue et al., 2013). Available data suggest a role for RhopH3/CLAG3.2 function in *Plasmodium* surface anion channel (PSAC), a new permeability pathway (NPP) generated in newly invaded merozoites and essential for allowing nutrient uptake during blood-stage development (Counihan et al., 2013). rama-rhoptry associated membrane antigen (RAMA) is thought to act as a molecular chaperone for other rhoptry proteins. It interacts with RAP1 and ClAG3.2, leading to the trafficking of these proteins to the nascent rhoptry (Richard et al., 2009). We speculate that the upregulation and interplay of these genes in SA-independent parasites suggest structural and physiological roles in signaling efficient rhoptry releases for fastening onto the RBC surface for alternative pathways of RBC invasion and immune evasion. This is in line with recent evidence for the upregulation of SURFIN and EPF protein members in the transcriptome profile of *P. falciparum* W2mef parasites that switch invasion phenotypes from SA-dependent to SA-independent pathways when grown under flow conditions (Nyarko et al., 2020).

Of equal importance was the significantly higher expression of genes encoding PfCDPK2, PfCDPK5, PfHP2, and PfDOC in the transcriptome profile of SA-dependent parasites relative to SA-independent parasites. An increase in the concentration of calcium ions in the bloodstream is known to trigger micronemal secretions of EBA ligands for binding GYPA and GYPC receptors, deforming the RBC membrane and making it more invasive. Microneme release of EBA 140 and EBA 175 has been shown to require calcium-dependent membrane–membrane interactions or transients that have been shown to be mediated by CDPK-dependent signaling (Absalon et al., 2018), phosphoinositide binding protein (PH2) (Ebrahimzadeh et al., 2019), and proteins with double C2 domains (Jean et al., 2014). Hence, this explains their upregulation in SA-dependent parasites that rely on EBA ligand–receptor interactions for invasion.

Another feature of the transcriptome profiling data was the high expression of genes encoding *P. falciparum* apicomplexan activator protein-2 (*Pf*AP2) transcription factors in SA-independent parasites. AP2-G2 transcription factor is epigenetically controlled and together with the duffy binding-like domain of merozoite surface protein2 (DBLMSP2), which was also upregulated, have been shown to be associated with gametocyte development(GDV1) in parasites committed to sexual development (Poran et al., 2017). A recent study has shown that AP2-G2 interacts with PfAP2-invasion (AP2-I) and *P. falciparum* bromodomain protein 1 (PfBDP1) to drive the expression of invasion-related genes (*msp1* and *ama1*) in sexually committed merozoites, potentially increasing their invasion efficiency (Josling et al., 2020). It can be speculated that the upregulation of these transcription factors is important for the efficient alternative RBC invasion pathways in sexually committed parasites to sustain parasite growth during blood-stage infection.

The absence of biological replicates (Schurch et al., 2016) and the inability to enrich for 100% schizont-stage cultures could be potential limitations of our schizont transcriptome DEG analysis. Nonetheless, our transcriptome data were rich (~85%) in schizont stages, and given the technical intricacy of achieving biological replicates with our cryopreserved clinical samples, we opted for a high sequencing depth (averaging 78.89X across all samples) and only focused our differential analysis on genes that are highly expressed as proposed elsewhere (Tarr et al., 2018). Yet, we cannot underscore the importance of a larger sample size that may have helped refine the study findings. Taken together, we have shown that endemic Cameroonian isolates predominantly utilize SA-independent pathways of RBC invasion and that the use of SA-independent RBC invasion pathways is associated with transcriptional variation in genes encoding proteins controlling merozoite organelle discharges and exported virulence proteins involved in host RBC remodeling. These proteins can be further explored for use as therapeutic or vaccine targets against endemic blood stage parasites.

## Data availability statement

The original contributions presented in the study are publicly available. This data can be found here: Gene Expression Omnibus (GEO), accession GSE264687.

## Ethics statement

The studies involving humans were approved by Faculty of Health Sciences, Institutional Review Board, University of Buea, Cameroon. The studies were conducted in accordance with the local legislation and institutional requirements. Written informed consent for participation in this study was provided by the participants’ legal guardians/next of kin.

## Author contributions

IN: Writing – review & editing, Writing – original draft, Visualization, Project administration, Methodology, Investigation, Funding acquisition, Formal analysis, Data curation, Conceptualization. KM: Writing – review & editing, Visualization, Validation, Software, Data curation. JM: Writing – review & editing, Resources, Methodology. FB: Writing – review & editing, Methodology. AJ: Writing – review & editing, Methodology. TA: Writing – review & editing, Validation, Supervision, Resources. DA: Writing – review & editing, Validation, Supervision, Resources. UD: Writing – review & editing, Validation, Resources, Project administration. AN: Writing – review & editing, Visualization, Validation, Supervision, Software, Resources, Project administration, Funding acquisition, Conceptualization.
