# Supplementary figures and images for: Transcriptome analysis reveals molecular targets of erythrocyte invasion phenotype diversity in natural Plasmodium falciparum isolates from Cameroon
Source: Front Parasitol. 2024 May 24;3:1370615. doi: 10.3389/fpara.2024.1370615 (PMC11731687; doi:10.3389/fpara.2024.1370615)

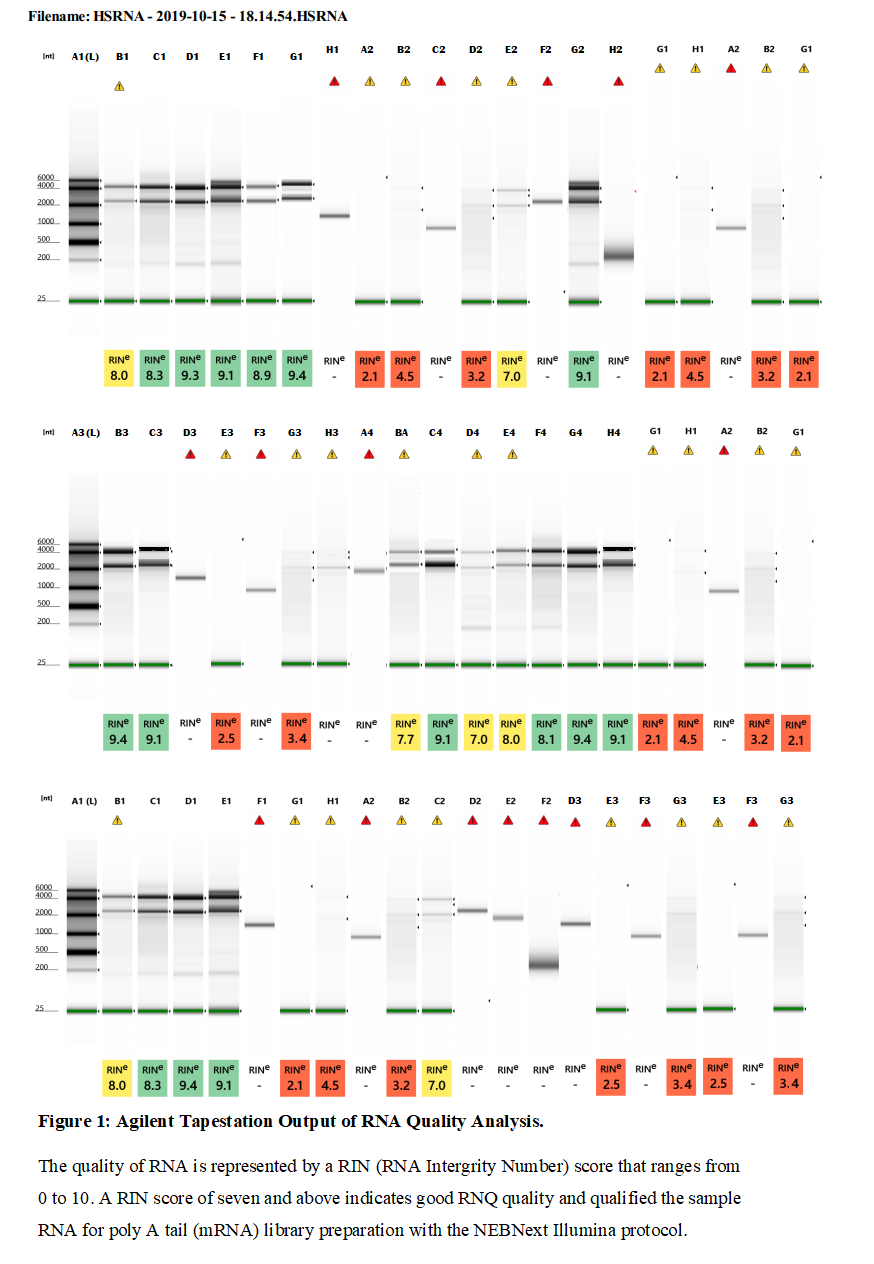

Supplement: Supplementary file 1 [file DataSheet_1.zip › Figure S1.PNG]

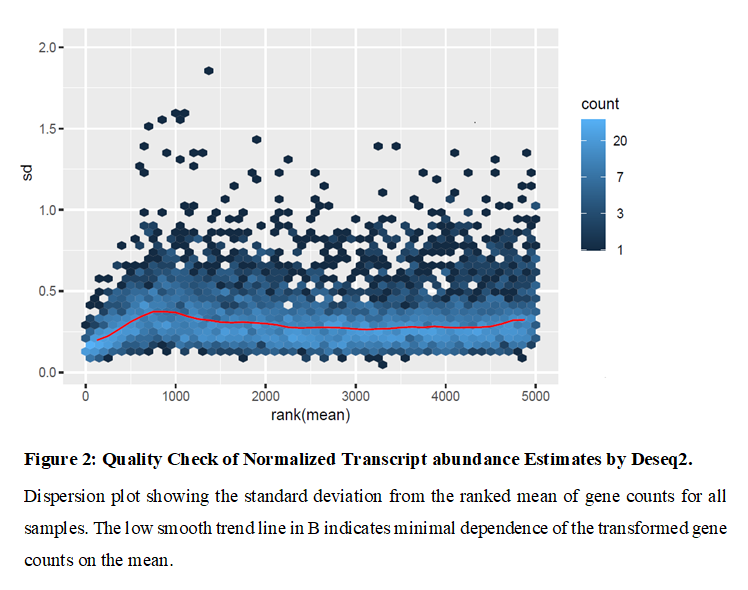

Supplement: Supplementary file 1 [file DataSheet_1.zip › Figure S2.PNG]

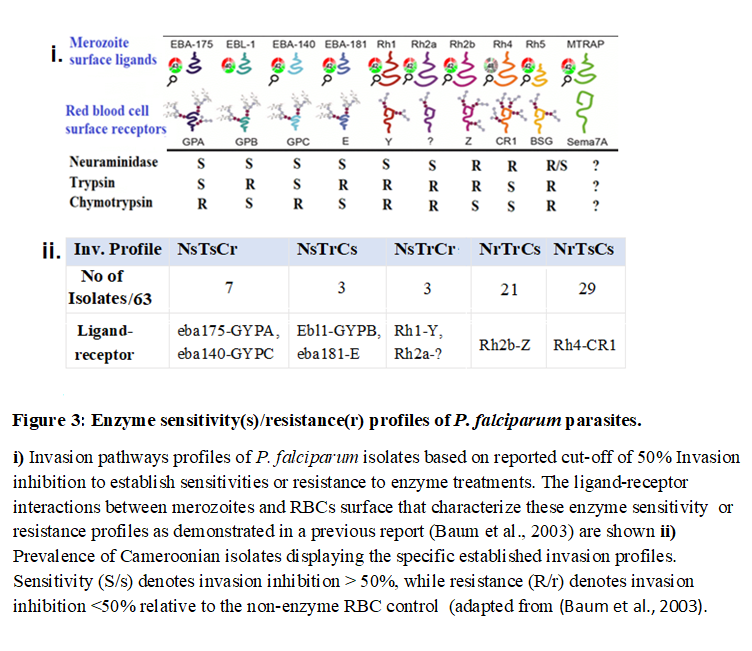

Supplement: Supplementary file 1 [file DataSheet_1.zip › Figure S3.PNG]

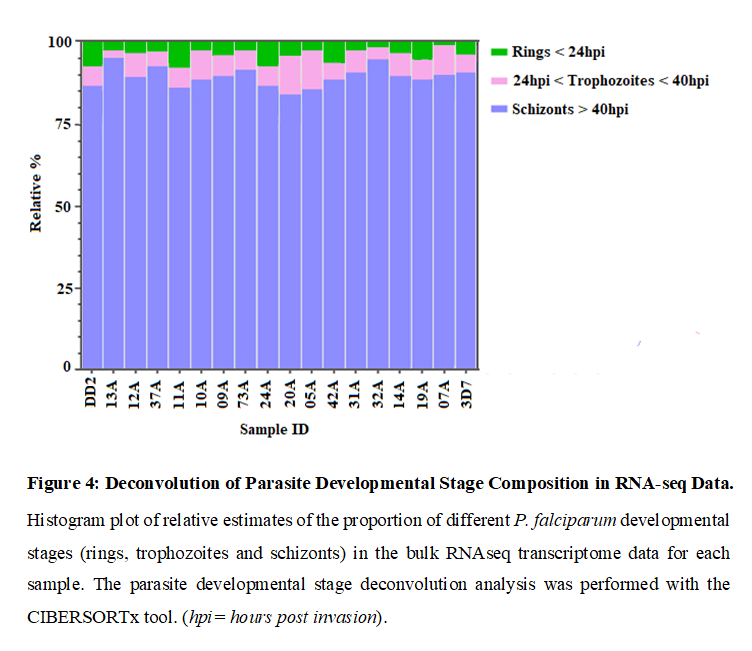

Supplement: Supplementary file 1 [file DataSheet_1.zip › Figure S4.PNG]

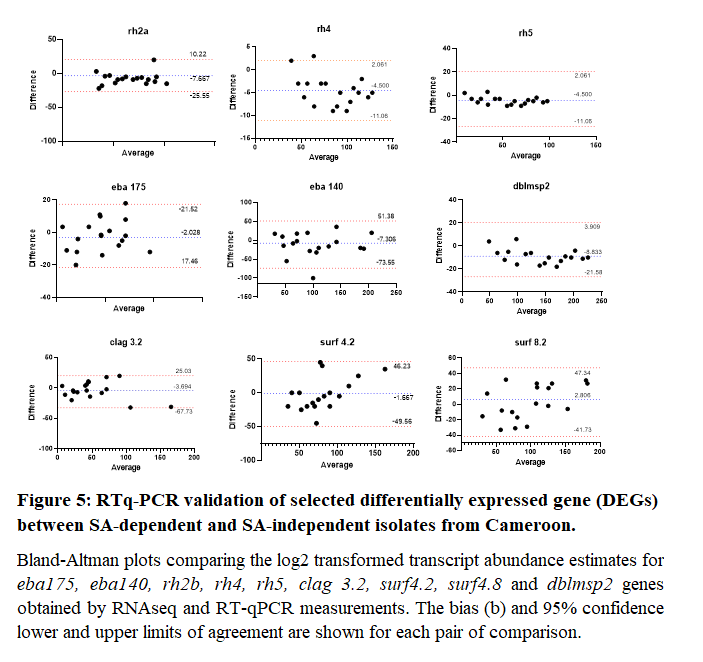

Supplement: Supplementary file 1 [file DataSheet_1.zip › Figure S5.PNG]
