# Supplementary material for: Transcriptome analysis reveals molecular targets of erythrocyte invasion phenotype diversity in natural Plasmodium falciparum isolates from Cameroon
Source: Front Parasitol. 2024 May 24;3:1370615. doi: 10.3389/fpara.2024.1370615 (PMC11731687; doi:10.3389/fpara.2024.1370615)
Supplement: Supplementary file 1 [file DataSheet_1.zip › Supplementary Material.DOC]

**Supplementary Material**

**Transcriptome Analysis Reveals Molecular Targets of Invasion Phenotype Diversity in Natural *Plasmodium falciparum* Isolates from Malaria Endemic Regions of Cameroon**

Ines A. Ngoh^1,3^, Karim Mane^2,3^, Jarrah Manneh^3^, Fatoumata Bojang^3^, Aminata S. Jawara^3^, Theresia N. Akenji^1^, Damian N. Anong^1^, Umberto D'Alessandro^3^, Alfred A. Ngwa^3*^

**1.** Flow Cytometry Data Acquisition and Gating Strategy for obtaining reinvasion parasitemia

Data was acquired within 24hours of SyBr Green 1 staining. Cell counting by flow cytometry was done with a BD Accuri™ C6 flow cytometer (BD Biosciences, Oxford, UK) equipped with a 488 nm 20 mW blue laser and a 633 nm 17 mW red laser for exciting SYBR Green1 (Invitrogen S7563) and CTFR Proliferation (Invitrogen C34564) dyes respectively. Initial data visualization and analysis were performed using BD6 Csampler software. Detectors of forward scatter-FSC, side scatter-SSC, FL1 (SyBr Green I) and FL4 (CTFR Prol) were set to logarithmic scale and 100,000 events were collected for each assay well.

The gating strategy used for quantifying reinvasion parasitemia is shown in the figure below. Briefly, intact RBCs were selected with a polygonal gate (P1) on an SSC/FSC plot (Figure i). Fluorescence of infected RBCs (iRBCs) was separated from RNA-containing uninfected RBCs (unRBCs) on a FL1/SyBr Green 1 histogram plot by setting cluster (gates) for iRBCs at 10 fluorescent units (FU) away from the peak of the unRBCs population. This excluded weak background fluorescence from RNA-containing unRBCs, previously shown to localize within this region with pico-green (Jun et al., 2012) and with SyBr Green (Malleret et al., 2011) (Figure ii). Gates for total iRBCs (R1) and labeled target RBCs (R2) were then set on corresponding FL1-H/SyBr Green 1 and FL4-H/CTFR Prol dot plots (Figures iii and iv respectively). The total iRBCs include iRBCs in both donor unlabeled and target labelled RBCs. To obtain reinvasion parasitemia, rather than the conventional gating of double positives (i.e CTFR and SBr Green 1 positive RBCs or infected labelled target RBCs), as Q1-UR on quadrant plots (Figure iv), we gated double positives (R3) from R1 and R2 events on bivariate dot plots of SyBr Green 1/ CTFR Prol (Figure v).

**2. Assessing the Proportion of Parasite Developmental Stages in the Transcriptome Data.**

The relative proportions of different parasite developmental stages will influence gene expression profiles. Although significant effort was made to ensure merozoite-containing schizont stage enrichment of parasite cultures, different parasite isolates and clones within complex isolates may have different growth rates, and may not always be synchronous. To assess the proportion of different parasite development stages in the transcriptome data, two approaches were employed.

Firstly, the gene counts or transcript abundance estimates in fragments per kilo bases per million mapped reads (FPKM), generated in DESeq2, of our transcriptome data were correlated (using Spearman’s rank correlation) with a reference file (Ott*o et a*l., 2010) containing FPKMs of single cell transcriptome harvested from seven cultured time points 0, 8, 16, 24, 32, 40 and 48 hours post invasion (hpi) over an ~ 48 h P. falciparum time course experiment.

The second approach involved using gene expression deconvolution analysis using the CIBERSORT v1.08 tool (Newma*n et a*l., 2015) as described elsewhere (Bradwel*l et a*l., 2020). Briefly, gene expression signature profiles (50-150 genes) corresponding to ring, trophozoite and schizont stages generated from a single cell RNA-seq experiment (Pora*n et a*l., 2017) was used as a reference input data to statistically separate the transcriptional signal of corresponding parasite developmental stages in our bulk RNA-seq data using the CIBERSORTx tool.

REFERENCES

Bradwell, K.R., Coulibaly, D., Koné, A.K., Laurens, M.B., Dembélé, A., Tolo, Y., Traoré, K., … Travassos, M.A. & Serre, D. (2020) ‘Host and Parasite Transcriptomic Changes upon Successive *Plasmodium falciparum* Infections in Early Childhood’, *mSystems*, Edited by P. Flórez de Sessions, 5, e00116-20, /msystems/5/4/msys.00116-20.atom.

Newman, A.M., Liu, C.L., Green, M.R., Gentles, A.J., Feng, W., Xu, Y., Hoang, C.D., Diehn, M. & Alizadeh, A.A. (2015) ‘Robust enumeration of cell subsets from tissue expression profiles’, *Nature methods*, 12, 453–457.

Otto, T.D., Wilinski, D., Assefa, S., Keane, T.M., Sarry, L.R., Böhme, U., Lemieux, J., Barrell, B., Pain, A., Berriman, M., Newbold, C. & Llinás, M. (2010) ‘New insights into the blood-stage transcriptome of Plasmodium falciparum using RNA-Seq’, *Molecular Microbiology*, 76, 12–24.

Poran, A., Nötzel, C., Aly, O., Mencia-Trinchant, N., Harris, C.T., Guzman, M.L., Hassane, D.C., Elemento, O. & Kafsack, B.F.C. (2017) ‘Single-cell RNA-seq reveals a signature of sexual commitment in malaria parasites’, *Nature*, 551, 95–99.
